# Canine Babesiosis in Northwestern India: Molecular Detection and Assessment of Risk Factors

**DOI:** 10.1155/2014/741785

**Published:** 2014-06-12

**Authors:** Amritpal Singh, Harkirat Singh, N. K. Singh, N. D. Singh, S. S. Rath

**Affiliations:** ^1^Department of Veterinary Parasitology, College of Veterinary Science, Guru Angad Dev Veterinary and Animal Sciences University, Ludhiana, Punjab 141 004, India; ^2^Department of Veterinary Pathology, College of Veterinary Science, Guru Angad Dev Veterinary and Animal Sciences University, Ludhiana, Punjab 141 004, India; ^3^Animal Disease Research Centre, College of Veterinary Science, Guru Angad Dev Veterinary and Animal Sciences University, Ludhiana, Punjab 141 004, India

## Abstract

In the current study, a total of 214 blood samples from dogs in and around Ludhiana, Punjab (India), suspected for canine babesiosis were examined with conventional and molecular assays. Examination of Giemsa-stained peripheral thin blood smears revealed an overall prevalence of 7.47% (16/214) for canine babesiosis encompassing 0.93% (2/214) of large *Babesia* and 6.54% (14/214) of *Babesia gibsoni*. However, molecular diagnosis revealed 15.42% (33/214) samples positive for *B. gibsoni* infection as evident by the presence of 671 bp amplicon. The results of multivariate analysis showed that the prevalence of *B. gibsoni* was associated with various risk factors, namely, age (*P* < 0.001; OR: 0.398; CI 95%: 0.080–1.799), sex (*P* = 0.022; OR: 0.849; CI 95%: 0.403–1.791), breed of host (*P* = 0.371; OR: 3.345; CI 95%: 1.045–10.710), and season (*P* = 0.230; OR: 2.143; CI 95%: 0.788–5.830). The prevalence of *B. gibsoni* was higher in summer as compared to winter season and in younger dogs, while breed and sex of the host were not significantly associated with the occurrence of the disease.

## 1. Introduction 

Amongst the various prevalent canine vector-borne diseases, canine babesiosis is very common and clinically significant disease caused by intraerythrocytic apicomplexan protozoa belonging to genus* Babesia*, distributed worldwide, including India.* Babesia *species often referred to as piroplasms comprise two main species,* B. canis *and* B. gibsoni, *based on their size.* B. canis *is a large piroplasm (4-5 *μ*m), which usually occurs as a single pear-shaped piroplasm or in pairs of merozoites divided by binary fission within the erythrocyte.

Previous studies, on the basis of differences in the geographical distribution, vector specificity, and antigenic properties [1, 2], recognized that large canine piroplasms are subdivided into three species, namely,* B. canis *transmitted by* Dermacentor reticulatus *(in Europe),* B. vogeli* transmitted by* Rhipicephalus sanguineus *(in tropical and subtropical regions), and* B. rossi* transmitted by* Haemaphysalis elliptica *(in South Africa).* B. gibsoni *has been found to be associated with infection of dogs in Asia, North America, northern and eastern Africa, and Europe [3–5]. It is a small parasite that commonly appears as individual ring forms or pyriform bodies ranging between 1.0 and 2.5 *μ*m in size [3].

Clinically canine babesiosis has been found to result in a wide range of presentations from subclinical disease to serious illness characterised by fever, pallor, jaundice, splenomegaly, weakness and collapse associated with intra- and extravascular haemolysis, hypoxic injury, systemic inflammation, thrombocytopenia, and pigmenturia [6].

As far as the diagnosis of canine babesiosis is concerned, direct microscopic examination of the stained blood smear is the most commonly used method as it is conclusive, feasible, and cost effective diagnostic method but not necessarily detects parasites in dogs with unapparent or chronic infections since the level of parasitemia is very low [7]. As regards, the serological methods, indirect fluorescent antibody test (IFAT) and enzyme linked immunosorbent assay (ELISA) for* B. gibsoni* parasites, are considered to be highly sensitive, but only moderately specific because of antigenic cross-reactions to* B. canis* [8] and normal dog erythrocytes [8, 9]. Therefore, the development of highly specific and sensitive system for the diagnosis of canine babesiosis is still awaited. In this regard, recent advances in molecular biology techniques like polymerase chain reaction (PCR) have made it possible to detect and identify piroplasms with greater sensitivity and specificity than traditional methods [10, 11].

Regarding Indian scenario, though there are sporadic reports of canine babesiosis based on conventional diagnostic methods [12–15], the true status of canine babesiosis is still not clear barring few reports [16, 17] employing the PCR based assays. Furthermore, molecular detection of canine babesiosis has not yet been explored from Punjab, north state of India, so the present work was carried out to know the status of canine babesiosis in this part of the country through PCR based assays.

## 2. Materials and Methods

### 2.1. Geographical Area

The study was conducted from Ludhiana district of Punjab state, in the northwestern region of India. The climate of the region under study is excessively hot and dry during summers. Winters are cool with some frosts, and the average annual rainfall is 565.9 mm. These environmental conditions provide favourable and conducive conditions for the survival and propagation of ticks and* Rhipicephalus sanguineus* is the major tick infesting canines [18].

### 2.2. Samples

A total of 214 blood samples were collected aseptically from cephalic vein of the selected dogs in EDTA coated vials from the dogs presented to Small Animal Clinics, Teaching Veterinary Clinical Complex, GADVASU, Ludhiana, as well as local private veterinary clinics from a period of one year (April 2012 to March 2013). Dogs were selected on the basis of presence of naturally acquired tick infestation at the time of presentation and/or showing clinical signs in accordance with the haemoprotozoan infection, namely, fever, haemoglobinuria, anemia, and so forth. The collected blood samples were utilized immediately for the preparation of thin blood smears and were then kept at −20°C until DNA extraction.

Microscopic examination of blood samples was done after staining the prepared thin blood smears with Giemsa as per standard protocol [19] and examined under oil immersion objective of the microscope to detect the piroplasms and the results obtained were compared to that of PCR assay.

### 2.3. Genomic DNA Isolation

For conducting the PCR assay, genomic DNA was isolated from whole blood using QIAamp DNA blood mini kit (QIAGEN, GmbH, Germany) following the manufacturer's recommendations with minor modifications and stored at −20°C till use. Genomic DNA of* B. gibsoni* was isolated and utilized as a positive control from infected blood sample showing parasitemia in blood smear examination. Genomic DNA was also isolated from the whole blood of infection-free puppy and used as a negative control along with nuclease-free water.

### 2.4. PCR Protocol

The PCR assay was optimized targeting a portion of the 18S rRNA gene to amplify* B. gibsoni* as described by Inokuma et al. [20]. The sequences of the primers were as follows:

 Gib599 Forward: 5′CTCGGCTACTTGCCTTGTC3′;
 Gib1270 Reverse: 5′GCCGAAACTGAAATAACGGC3′.

PCR assay in a final volume of 25 *μ*L was carried out in a PCR thermal cycler (Applied Biosystems, USA). The master mix consisted of 2.5 *μ*L of 10X PCR buffer (MBI Fermentas), 0.5 *μ*L of 10 mM dNTP mix (MBI Fermentas), 1.5 *μ*L of 25 mM MgCl_2_ (MBI Fermentas), 1.0 U of recombinant Taq DNA polymerase (MBI Fermentas), 1 *μ*L each (20 pmol) of the primers, and 5 *μ*L of template DNA isolated from field samples. The volume was made up to 25 *μ*L with nuclease-free water. The PCR cycling conditions were initial denaturation at 95°C for 5 min, 40 cycles of denaturation at 95°C for 30 sec, annealing at 56°C for 30 sec, and extension at 72°C for 1:30 min, and the final extension was performed at 72°C for 5 min. The PCR products obtained were checked for amplification by electrophoresis on a 1.5% agarose gel and visualized using gel documentation system (Syngene, UK). In order to check the specificity of the assays, isolated genomic DNA of large* Babesia*,* Ehrlichia canis*,* Hepatozoon canis,* and* Trypanosoma evansi* isolated from the microscopically positive cases were also employed in the PCR to see the amplification, if any.

### 2.5. Statistical Analysis

All data analyses were performed by using statistical software program (SPSS for Windows, Version 19.0, USA). Association between the prevalence of* B. gibsoni* by PCR and various risk factors, namely, sex, age, breed of the host, and season, was carried out by Chi square (*χ* ^2^-test). Variables with significant association at *P* < 0.05 (two-sided) were subjected to the multivariate logistic regression model. The results were each expressed as *P* value and odds ratio (OR) with a 95% confidence interval (CI 95%).

## 3. Results

### 3.1. Blood Smear Examination

In the present study, examination of Giemsa-stained peripheral thin blood smears of 214 canines revealed an overall prevalence of canine babesiosis as 7.47% with 0.93% (2/214) positivity for the piroplasms of large* Babesia* and 6.54% (14/214) positivity for the piroplasms of* B. gibsoni*.

### 3.2. PCR Protocol

**Figure 1:**
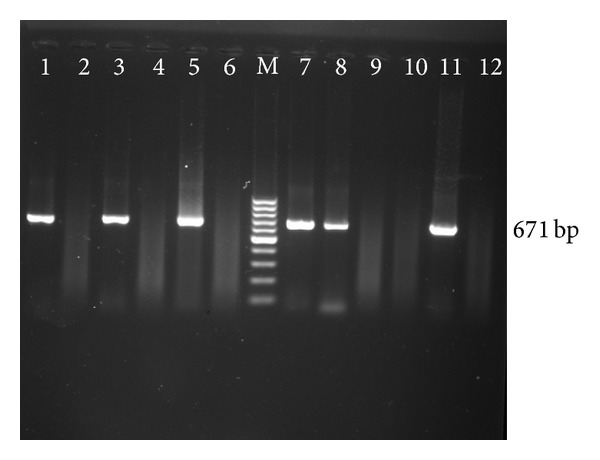
Gel electrophoresis showing* B. gibsoni* PCR assay. Lane M: GeneRuler 100 bp Ladder, lanes 1–5 and 8–12: field collected samples, lane 6: negative control, and lane 7: positive control.

All the collected blood samples were analyzed by PCR assay, to detect any amplification in the form of ethidium bromide-stained amplicons, after standardization. Of the total samples subjected 15.42% (33/214) were positive for presence of* B. gibsoni* infection as revealed by the amplification of a 671 bp product (Figure 1). Further, the PCR primers used in the present assay did not amplify any product when the genomic DNA of large* Babesia*,* E. canis*,* H. canis*, and* T. evansi* were used as template revealing the specificity of these primers. The sensitivity, specificity, and diagnostic efficacy of PCR were determined by using blood smear examination as the gold standard and results are presented in Table 1.

### 3.3. Correlation of Canine Babesiosis with Various Risk Factors

The correlation between the prevalence of* B. gibsoni* and various risk factors was studied and the values of the correlation coefficients (*β*) are presented in Table 2. Further, the results of multivariate analysis showed that the prevalence of* B. gibsoni* was associated with various risk factors, namely, age (*P* < 0.001; OR: 0.398; CI 95%: 0.080–1.799), sex (*P* = 0.022; OR: 0.849; CI 95%: 0.403–1.791), breed of host (*P* = 0.371; OR: 3.345; CI 95%: 1.045–10.710), and season (*P* = 0.230; OR: 2.143; CI 95%: 0.788–5.830). The prevalence of* B. gibsoni* was higher in summer as compared to winter season and in younger dogs, while breed and sex of the host were not significantly associated with the occurrence of the disease (Table 3).

## 4. Discussion

In the present study by using conventional parasitological techniques, a statistically higher percent positivity was recorded for* B. gibsoni* infection than large* Babesia* (*P* = 0.0051) with the overall prevalence of canine babesiosis as 7.47% (16/214). Previously, from the same region, Eljadar [21] examined a total of 951 suspected dog samples from Small Animal Clinics, GADVASU, Ludhiana, and three local private veterinary hospitals for haemoprotozoan infections and reported 1.26% samples to be positive for* B. canis *and 3.17% to be positive for* B. gibsoni*. The comparative higher prevalence of* B. gibsoni* over* B. canis* recorded by him is in congruence with that of the present study. Similar findings were recorded in earlier studies by Singh et al. [15, 22] from this region revealing the prevalence of* B. gibsoni *and* B. canis *in the range of 0.65%–8.26% and 1.43%–4.51%, respectively.

Microscopic detection of* B. gibsoni*, though smaller in size than large* Babesia*, was easier because of its frequent appearance in the circulating host blood. This might also be due to a low level parasitaemia in case of large* Babesia* infection especially during very early or carrier stage which is beyond the level of microscopic detection [6, 10, 23]. The prevalence of canine babesiosis from various parts of northern India has been reported to be ranging from 0.66 to 8.9% [14, 22, 24, 25] while from Southern India Senthil Kumar et al. [13] recorded 3.9% and 84.9% prevalence of* B. canis* and* B. gibsoni*, respectively. Wide variation in climatic conditions prevailing in different parts of India might be responsible for varying percentage of these tick borne infections.

On the basis of present findings PCR based assay was able to detect 15.42% prevalence of* B. gibsoni*. Higher detection of canine babesiosis by PCR based assays as compared to microscopy as observed in the present study has also been reported by several authors worldwide indicating the higher sensitivity levels of PCR [10, 17, 26–30]. As far as the detection of* B. gibsoni* with PCR based assays is concerned, many studies have been carried out worldwide and the prevalence has been recorded to be ranging from 3.3 to 55% [17, 20, 31–34].

As far as evaluation of various risk factors is concerned for canine babesiosis, several authors have observed the prevalence of the haemoprotozoan infections to be highest in young dogs [35, 36]. In terms of sex of the host, from the data obtained in the current study, it can be concluded that the assays recorded no statistical significance difference in the prevalence of the disease among males and female dogs. These results are incongruous with Amuta et al. [28] and Singh et al. [14].

Regarding breed of the host, the results revealed that blood smear examination and PCR detected a statistically nonsignificant difference in the prevalence of the* B. gibsoni* among the various breeds and nondescript dogs. In seasonal prevalence of the disease, the disease was most prevalent in warm seasons as compared to winters. The probable reason behind this trend may be correlated to the seasonal activity of the brown dog tick,* Rhipicephalus sanguineus* which is in its abundance in hot and humid period of the year, thus resulting in the higher incidence of haemoprotozoan infections in warm months during warmer seasons [37].

## Figures and Tables

**Table 1:** Evaluation of diagnostic/screening PCR assays over blood smear examination.

Parameter	PCR (95% CI)
Sensitivity*	100% (78.47, 100)
Specificity*	90.5% (85.64, 93.83)
Diagnostic accuracy*	91.12% (86.55, 94.24)

*Wilson score (http://www.openepi.com/v37/DiagnosticTest/DiagnosticTest.htm).

**Table 2:** Final logistic regression model for factors associated with prevalence of *B. gibsoni* by PCR on animal levels.

Variable	Regression coefficient (*β*)	Standard error (SE)	*P* value	Odds	CI (95%)*
Age	−1.330	0.199	0.000	0.398	0.080–1.799
Sex	0.346	0.151	0.022	0.849	0.403–1.791
Breed	0.179	0.200	0.371	3.345	1.045–10.710
Season	−0.223	0.186	0.230	2.143	0.788–5.830

*Confidence interval.

**Table 3:** Assessment of various risk factors with regard to distribution of *B. gibsoni* infection.

Risk factor	Parameter	Number	Blood smear (%)	PCR (%)
Age	0–6 m	34	2 (5.88)	2 (5.88)
	6 m–1 y	40	6 (15)	12 (30)
	>1 y	140	6 (4.28)	19 (13.57)
	*χ* ^2^ value		4.653	8.543*

Sex	Male	124	8 (6.45)	18 (14.51)
	Female	90	6 (6.67)	15 (16.67)
	*χ* ^2^ value	—	0.066	0.246

Breed	Labrador	71	7 (9.85)	16 (22.53)
	German Shepherd	34	3 (8.82)	8 (23.52)
	Pug	24	1 (4.16)	2 (8.33)
	Others	35	—	3 (8.57)
	Nondescript	50	3 (6)	4 (8)
	*χ* ^2^ value	—	5.201	7.829

Season	Summer	64	5 (7.81)	12 (18.75)
	Rainy	78	6 (7.69)	14 (17.94)
	Winter	72	3 (4.16)	7 (9.72)
	*χ* ^2^ value	—	0.004	1.827

Total		214	14 (6.54)	33 (15.42)

*P < 0.05; others include Pomeranian (8), Saint Bernard (9), Dalmatian (3), Boxer (3), Great Dane (3), Cocker Spaniel (2), Rottweiler (4), and Napoleon Mastiff (2).

## References

[1] Uilenberg G, Franssen FFJ, Perié NM, Spanjer AAM (1989). Three groups of *Babesia canis* distinguished and a proposal for nomenclature. *Veterinary Quarterly*.

[2] Hauschild S, Shayan P, Schein E (1995). Characterization and comparison of merozoite antigens of different *Babesia canis* isolates by serological and immunological investigations. *Parasitology Research*.

[3] Conrad PA, Thomford J, Yamane I (1991). Hemolytic anemia caused by *Babesia gibsoni* infection in dogs. *Journal of the American Veterinary Medical Association*.

[4] Casapulla R, Baldi L, Avallone V, Sannino R, Pazzanese L, Mizzoni V (1998). Canine piroplasmosis due to *Babesia gibsoni*: clinical and morphological aspects. *Veterinary Record*.

[5] Birkenheuer AJ, Levy MG, Savary KCM, Gager RB, Breitschwerdt EB (1999). *Babesia gibsoni* infections in dogs from North Carolina. *Journal of the American Animal Hospital Association*.

[6] Irwin PJ (2009). Canine babesiosis: from molecular taxonomy to control. *Parasites and Vectors*.

[7] Cacciò SM, Antunovic B, Moretti A (2002). Molecular characterisation of *Babesia canis* canis and *Babesia canis vogeli* from naturally infected European dogs. *Veterinary Parasitology*.

[8] Yamane I, Conrad PA, Gardner IA (1993). *Babesia gibsoni* infection in dogs. *Journal of Protozoology Research*.

[9] Adachi K, Tateishi M, Horii Y, Nagatomo H, Shimizu T, Makimura S (1994). Reactivity of serum anti-erythrocyte membrane antibody in *Babesia gibsoni*-infected dogs. *The Journal of Veterinary Medical Science*.

[10] Birkenheuer AJ, Levy MG, Stebbins M, Poore M, Breitschwerdt E (2003). Serosurvey of anti-*Babesia* antibodies in stray dogs and American pit bull terriers and American Staffordshire terriers from North Carolina. *Journal of the American Animal Hospital Association*.

[11] Jefferies R, Ryan UM, Muhlnickel CJ, Irwin PJ (2003). Two species of canine *Babesia* in Australia: detection and characterization by PCR. *Journal of Parasitology*.

[12] Sundar N, Balachandran C, Senthivelan A (2004). Incidence of *Babesia gibsoni* infection in dogs in Tamil Nadu. *Journal of Veterinary Parasitology*.

[13] Senthil Kumar K, Vairamuthu S, Kathiresanl D (2009). Prevalence of haemoprotozoans in canines in Chennai City. *Tamilnadu Journal of Veterinary and Animal Science*.

[14] Singh NK, Haque JM, Singh H, Rath SS (2011). Prevalence of canine parasitic infections. *Indian Veterinary Journal*.

[15] Singh H, Haque M, Jyoti, Singh NK, Rath SS (2012). Occurrence of parasitic infections in dogs in and around Ludhiana, Punjab (India). *Applied Biological Research*.

[16] Abd Rani PAM, Irwin PJ, Coleman GT, Gatne M, Traub RJ (2011). A survey of canine tick-borne diseases in India. *Parasites and Vectors*.

[17] Laha R, Bhattacharjee K, Sarmah PC (2013). *Babesia* infection in naturally exposed pet dogs from a north-eastern state (Assam) of India: detection by microscopy and polymerase chain reaction. *Journal of Parasitic Diseases*.

[18] Gill HS, Gill BS (1977). Qualitative district-wise distribution of adult ixodid ticks in the Punjab state. *Ixodid Ticks of Domestic Animals in the Punjab State*.

[19] Coles EH (1986). *Veterinary Clinical Pathology*.

[20] Inokuma H, Yoshizaki Y, Matsumoto K (2004). Molecular survey of *Babesia* infection in dogs in Okinawa, Japan. *Veterinary Parasitology*.

[21] Eljadar MSM (2010). *Clinico-diagnostic studies on vector transmitted haemoprotozoan diseases in dogs [M.S. thesis]*.

[22] Singh H, Haque M, Singh NK, Rath SS (2011). Prevalence of canine parasitic infections in and around Ludhiana, Punjab. *Journal of Veterinary Parasitology*.

[23] Bourdoiseau G (2006). Canine babesiosis in France. *Veterinary Parasitology*.

[24] Varshney JP, Dey S (1998). A clinical study on haemoprotozoan infections in referral canines. *Journal of Remount and Veterinary Corps*.

[25] Chaudhuri S (2006). *Studies on clinico-therapeutic aspects of babesiosis in dogs [M.S. thesis]*.

[26] Götsch S, Leschnik M, Duscher G, Burgstaller JP, Wille-Piazzai W, Joachim A (2009). Ticks and haemoparasites of dogs from Praia, Cape Verde. *Veterinary Parasitology*.

[27] O’Dwyer LH, Lopes VVA, Rubini AS, Paduan KDS, Ribolla PEM (2009). *Babesia* spp. infection in dogs from rural areas of São Paulo State, Brazil. *Revista Brasileira de Parasitologia Veterinaria*.

[28] Amuta EU, Atu BO, Houmsou RS, Ayashar JG (2010). *Rhipicephalus sanguineus* infestation and *Babesia canis* infection among domestic dogs in Makurdi, Benue State-Nigeria. *International Journal of Academic Research*.

[29] Ionita M, Mitrea IL, Pfister K, Hamel D, Buzatu CM, Silaghi C (2012). Canine babesiosis in Romania due to *Babesia canis* and *Babesia vogeli*: a molecular approach. *Parasitology Research*.

[30] Matsuu A, Ono S, Ikadai H (2005). Development of a SYBR green real-time polymerase chain reaction assay for quantitative detection of *Babesia gibsoni* (Asian genotype) DNA. *Journal of Veterinary Diagnostic Investigation*.

[31] Mokhtar AS, Lim SF, Tay ST (2013). Molecular detection of *Anaplasma platys* and *Babesia gibsoni* in dogs in Malaysia. *Tropical Biomedicine*.

[32] Talukder MH, Matsuu A, Iguchi A, Roy BC, Nishii N, Hikasa Y (2012). PCR-based survey of vector-borne pathogens in dogs in Dhaka, Bangladesh. *Journal of Bangladesh Agricultural University*.

[33] Fukumoto S, Xuan X, Shigeno S (2001). Development of a polymerase chain reaction method for diagnosing *Babesia gibsoni* infection in dogs. *Journal of Veterinary Medical Science*.

[34] Macintire DK, Boudreaux MK, West GD, Bourne C, Wright JC, Conrad PA (2002). *Babesia gibsoni* infection among dogs in the southeastern United States. *Journal of the American Veterinary Medical Association*.

[35] Abdullahi SU, Mohammed AR, Trimnell A, Sannusi R, Alafiatayo R (1990). Clinical and haematological findings in 70 naturally occurring cases of canine babesiosis due to *Babesia canis*. *Journal of Small Animal Practice*.

[36] Samradhni D, Maske DK, Shobha R, Shinde PN (2005). Bionomics and haemodynamics in blood protozoal infections in dogs from Nagpur. *Indian Journal of Animal Health*.

[37] Soulsby EJL (1982). *Helminths, Arthropods and Protozoa of Domesticated Animals*.

